# Psychosocial Work Environment and Well-Being of Direct-Care Staff Under Different Nursing Home Ownership Types: A Systematic Review

**DOI:** 10.1177/07334648221131468

**Published:** 2022-10-09

**Authors:** Tomas Lindmark, Maria Engström, Sven Trygged

**Affiliations:** 1 Faculty of Health and Occupational Studies, Department of Social Work, 560570 University of Gävle, Gävle, Sweden; 2 Faculty of Health and Occupational Studies, Department of Caring Science, 560570 University of Gävle, Gävle, Sweden

**Keywords:** caregiving, health outcomes, long-term services and supports, marketization, working conditions

## Abstract

This systematic review investigated the psychosocial work environment and well-being of direct-care staff under different nursing home ownership types. Databases searched: Scopus, Web of Science, Cinahl, and PubMed, 1990–2020. Inclusion criteria: quantitative or mixed-method studies; population: direct-care staff in nursing homes; exposure: for-profit and non-profit ownership; and outcomes: psychosocial work environment and well-being. In total, 3896 articles were screened and 17(*n* = 12,843 participants) were assessed using the Joanna Briggs Institute Critical Appraisal tools and included in the narrative synthesis. The results were inconsistent, but findings favored non-profit over for-profit settings, for example, regarding leaving intentions, organizational commitment, and stress-related outcomes. There were no clear differences concerning job satisfaction. Job demands were higher in non-profit nursing homes but alleviated by better job resources in one study. The result highlights work environment issues, with regulations concerning for-profit incentives being discussed in terms of staff benefits.

**What this paper adds**

• A systematic review with a narrative synthesis of the literature, including 17 studies of the psychosocial work environment and well-being of direct-care staff under different nursing home ownership types.
• The review problematizes the rise of for-profit incentives in nursing homes concerning staff work environment and well-being and supplements existing research indicating that profit incentives may cause adverse outcomes for both staff and residents.

**Applications of study findings**

• Further welfare policy regulations may be needed concerning for-profit incentives, for example, addressing better-regulated work environments, ensuring that no costs are cut concerning job resources, and improving factors such as job control, for the benefit of both staff and residents.
• Additional longitudinal comparisons and evaluations of the work environment in for-profit and non-profit nursing homes are essential.

## Introduction

The nursing home workforce is a widely scrutinized issue, and a recent European Commission report addressed the labor shortages and tough working conditions in the sector. Access to nursing homes is inadequate in most of Europe, partly because of changes in ownership and labor structures (European Commission; Directorate-General for Employment; Social Affairs and Inclusion, 2021). A global shortage of healthcare workers is expected by 2030 (Liu et al., 2017), and population aging is a universal predicament (OECD, 2020), increasing the number of people who will require nursing home care. Simultaneously, the strong marketization trend in social welfare policies has led to an increase in the for-profit ownership of nursing homes in Europe (Harrington et al., 2017; Meagher et al., 2013) and the United States (Choiniere et al., 2016). Marketization reforms have led to policy and legislative “blind spots” with potential consequences for residents, nursing home staff, and the overall effectiveness of social and health services (Meagher et al., 2013). U.S. government-owned nursing homes decreased in number between 2003 and 2020 in favor of for-profit facilities (Kaiser Family Foundation, 2020). For-profit incentives have also been linked to reduced staffing levels in U.S. nursing homes (Harrington et al., 2012) and fewer hours of care in Canada (Hsu et al., 2016). Furthermore, subcontracting nursing homes to for-profit third-party agencies was described by interviewed Canadian direct-care staff as detrimental to their working environment (Banerjee et al., 2021). Private for-profit ownership was, in an umbrella review, associated with poorer results concerning health-related outcomes, specifically care quality in nursing homes (Herrera et al., 2014). A systematic review found that contracting out ownership was overall harmful to the work environment and job satisfaction of public service staff (Vrangbæk et al., 2015). Consequently, type of ownership seems to be an important factor to consider when evaluating nursing homes’ work environment.

### Type of Ownership

Nursing homes may be owned or managed by entities or providers with different ownership structures, such as public (i.e., government and quasi-government), non-profit, and for-profit entities. *Public ownership* is common in European countries, where nursing homes may be controlled by municipal governments or health departments (Feltenius, 2017; Meagher et al., 2013). *Non-profit ownership* occurs via non-profit entities, and any excess revenue is used to benefit nursing home residents. In *for-profit ownership*, facilities are owned and operated as businesses with the assumption that revenue may be collected by the owners or shareholders. In some countries, the control of nursing homes is delegated to external providers, meaning that public authorities or municipalities may delegate nursing home control to private actors in exchange for public funds (Feltenius, 2017). Both for-profit and non-profit nursing homes may receive public or private funding.

Previous research has investigated the impact of profit incentives on factors such as quality of care and resident well-being. In a systematic review and meta-analysis, Comondore et al. (2009) found that non-profit nursing homes provided higher average quality of care than for-profit ones, especially in U.S. nursing homes. Similar results were found by Ronald et al. (2016) in a review of observational studies in Europe, North America, and Oceania. Another systematic review examined U.S. nursing homes, finding that non-profit facilities offered residents better overall quality of life than for-profit ones (Xu et al., 2013). Regarding work well-being, a systematic review by Bos et al. (2017) indicated that U.S. for-profit nursing homes tend to emphasize financial performance at the expense of work well-being and possibly resident well-being. They argued that to perform better financially, some for-profit nursing homes neglect work well-being and salaries to increase profits; however, their main measure of work well-being was staffing levels, which may not necessarily be equivalent to work well-being.

### Psychosocial Work Environment and Well-Being

Psychosocial work environment is a holistic concept that can be understood as an individual’s interaction with various organizational, psychological, and social components of everyday working life (Thylefors, 2015). The concept has been used in different job-strain models (Rugulies, 2019). The job demands-resources theory integrates several of these components in an attempt to emphasize that psychosocial factors (e.g., job demands) and resources may lead to negative outcomes such as burnout or positive outcomes such as work engagement, depending on the balance between demands and resources (Bakker & Demerouti, 2017). The Copenhagen Psychosocial Questionnaire (COPSOQ), an instrument based on this and other theories, lays out the psychosocial work environment in several domains (Burr et al., 2019).

Cooper et al. (2009) stated that well-being represents employee health in three forms: physical, mental, and emotional. Well-being as a concept may include indicators such as mental health, stress, job motivation, organizational commitment, job satisfaction, and intention to leave. It may also include depressive symptoms and work engagement (Burr et al., 2019). The present study utilized these definitions as a conceptual framework capturing the psychosocial work environment and well-being.

Previous studies have highlighted psychosocial work environment issues and work well-being among nursing home staff. A vicious cycle has been noted in which higher job demands lead to higher leaving intentions among direct-care staff (Van Aerschot et al., 2021), which may increase turnover rates. Job dissatisfaction and burnout syndromes are also common among nursing home staff (Cooper et al., 2016; White et al., 2019, 2020). Furthermore, an inadequate work environment among direct-care staff has been associated with deficient care for older adults (White et al., 2019). Similarly, a relationship has been observed between staff ratings of working life and older persons’ ratings of care quality (Engström et al., 2021; Lundgren et al., 2020). Considering previous research, for-profit incentives may be a factor that causes deficiencies in the psychosocial work environment.

In sum, the relationship between ownership type and care quality has been investigated in several systematic reviews (Comondore et al., 2009; Herrera et al., 2014; Xu et al., 2013), as have ownership and staffing levels (Bos et al., 2017) and the impact of subcontracting on working conditions in the public sector (Vrangbæk et al., 2015). However, the psychosocial work environment and well-being of nursing home staff have not been fully scrutinized in a systematic review. Furthermore, no completed or ongoing systematic reviews were found concerning nursing home ownership and staff well-being in the international prospective register of systematic reviews (PROSPERO) (NHS, 2018). This systematic review accordingly aimed to investigate the psychosocial work environment and well-being of direct-care staff under different nursing home ownership types. The results may suggest whether a profit orientation is problematic for the psychosocial work environment and staff well-being in nursing homes, affecting policies that could improve staff and residents’ quality of life.

## Methods

This systematic review was based on the Preferred Reporting Items for Systematic Reviews and Meta-Analyses (PRISMA) guidelines (Moher et al., 2009) and used sub-headings from the PRISMA checklist. While the article was being written, the PRISMA 2020 statement was released (Page et al., 2021), leading to additional topics for consideration. The population, exposure, and outcome (PEO) version of the population, intervention, comparator/s, and outcome (PICO) framework was used to guide the search process and to establish inclusion and exclusion criteria. The PEO framework is similar to PICO but focuses on associations between exposures rather than interventions and outcomes (Moola et al., 2020).

### Protocol and Registration

A PROSPERO protocol [CRD42020178775] (NHS, 2018) was established to outline the systematic review. This protocol was based on a previous search protocol formulated by the research group with help from an academic information specialist. The original protocol was formulated in January 2020 and completed in March 2020, whereas the PROSPERO protocol was formulated between March and June 2020.

### Eligibility Criteria

The PEO framework was used to identify the population (direct-care staff in nursing homes) as well as the exposure or risk factor (ownership type) and its outcomes (psychosocial work environment and well-being). The population comprised staff working at nursing homes involved in the direct care or service of residents: registered nurses, licensed practical nurses, nursing assistants, and nursing aides. Studies of nursing home residents or other staff not directly involved in care (e.g., janitors) were excluded. Nursing homes were also regarded as a population and comprised facilities where staff work around the clock to provide direct care (Table 1). Other types of health facilities and living arrangements (e.g., hospitals and hospices) and studies of exclusively home care nursing were excluded.

**Table 1.** Overview and Search Terms for the Electronic Search Using the Scopus Database.

No	Description		Items Found (Approx.)
**1**	Ownership	TITLE-ABS-KEY ((corporatization) OR (for-profit) OR (for profit) OR (owner*) OR (Proprietary) OR (“informal sector”) OR (“investor owned”) OR (non-profit) OR (non-profit) OR (“private enterprise”) OR (“private sector”) OR (“private care”) OR (privatization) OR (“profit orientation”) OR (“public care”) OR (“public sector”) OR (“public enterprise”) OR (investor) OR (“private* sponsor*”))	509 308
**2**	Nursing homes	TITLE-ABS-KEY (( “care setting”) OR (“continuing care”) OR (“older people care”) OR (“health services for the aged”) OR (“homes for the aged”) OR (“housing for the older people”) OR (“nursing facilities”) OR (“nursing homes”) OR (“old age homes”) OR (“retirement centers”) OR (“convalescent home”) OR (“assisted living”) OR (“long term care*") OR (“residential homes”))	289 427
**3**		1 AND 2	4356
**4**		3 AND Filters activated: Language (English, Swedish, Danish, Norwegian); time: 1990-; publication type: Article	2847 (Duplicates not removed)

The exposure was the ownership type: for-profit, non-profit, public, government, or private sector ownership; the risk factor associated with ownership type was profit incentives. Therefore, this review distinguished and grouped ownership types in terms of profit orientation rather than specific ownership types. A nursing home was considered for-profit based on whether it operated based on profit incentives (e.g., private for-profit nursing homes and nursing homes contracted out to for-profit providers). Non-profit nursing homes were those not based on profit incentives, such as nursing homes operated by the government, municipalities, or private non-profit organizations. In the result, government-owned facilities were grouped with non-profit nursing homes because neither have profit incentives. An additional criterion was that the included studies investigated differences between for-profit and non-profit nursing homes.

The outcomes comprised psychosocial work environment variables (e.g., job demands, job resources, job control, and social support) and well-being (e.g., stress, organizational commitment, turnover intention, and job satisfaction). Self-rated health was also included among well-being outcomes because of its strong link with psychosocial work environment variables and well-being in previous frameworks (Burr et al., 2019). Studies emphasizing work-related injuries, managers’ work environments, or managers’ perspectives were excluded.

Regarding study design, quantitative and mixed-method studies were considered. Systematic reviews, qualitative studies, editorials, discussion or opinion papers, commentaries, and non-empirical research were excluded. Qualitative studies were excluded because the aim was to investigate relationships between profit incentives and staff outcomes. The systematic search was limited to peer-reviewed articles in English, Swedish, Danish, and Norwegian published between January 1990 and March 2020. This timeframe was chosen to focus on the expansion of for-profit nursing homes and to set boundaries on the search strategy.

### Information Sources

Databases used to find articles were Scopus, Pubmed, Web of Science, and Cinahl. The search process was modified based on each database’s syllabus. The Scopus search strategy was originally developed and subsequently adapted to the syntax and subject headings of other databases. The PubMed search used MeSH terms and free-text words related to the review’s aim. Preliminary searches were overseen by the research group with assistance from the academic information specialist in January–February 2020. The preliminary search helped screen the search criteria; it showed that including both psychosocial work environment and well-being in the search string limited the relevant findings. The final search of all four databases was conducted on 30 March 2020 by the information specialist.

### Search Strategy

The search process was conducted in each database using search strings addressing two key concepts from the PEO framework: (a) *ownership*, including words such as “for-profit” and “public sector” AND (b) *nursing homes*, including words such as “older people care” and “nursing homes.” The intention was to use a broad search string to avoid missing important findings concerning psychosocial work environment or well-being variables. Four articles identified through the preliminary search were used as standard validation articles since they concerned nursing home ownership. If the search string for each database could identify this set of eligible studies, the search was considered valid. The following four articles were used as the validation set: Choi et al. (2012), Hamann and Foster (2014), Heponiemi et al. (2011), and Wendsche et al. (2016).

### Selection Process

The identified articles were imported into Rayyan software (Ouzzani et al., 2016) for continued screening and evaluation. Duplicates were removed using Rayyan. Two of the authors, independently of each other, reviewed the titles and abstracts in Rayyan to identify eligible studies that met the inclusion criteria; disagreements were resolved by discussion between these authors.

After the titles and abstracts were screened, the full-text articles that met the inclusion criteria were extracted and assessed by two independent reviewers (the first and last authors) to ensure that they fulfilled the eligibility criteria. Disagreements were resolved by a third reviewer. The reference lists of the included articles were manually searched for missed articles that met the inclusion criteria.

### Data Collection Process

Data were extracted by the first author based on the data items specified below and checked by the last author for errors. In three cases, the authors of the included articles were contacted through email to verify that the sample comprised nursing homes or to obtain further details on the type of ownership described in the study.

### Data Items

Descriptive data extracted from each selected study concerned study details (study date, title, author, and research aim), method and study setting (study design, country, year of data collection, ownership types considered, and inclusion/exclusion criteria), sample demographics (number of participants, gender, age, staff mix, and response rate), variables investigated (exposure, primary outcome, potential confounders, and how these were measured), outcomes (outcome name and types, and how outcomes were measured/reported), and relevant statistical results.

### Risk of Bias Assessment

The risk of bias was evaluated by two independent reviewers (first and last authors) using the Joanna Briggs Institute (JBI) Critical Appraisal tools (Briggs, 2014). Disagreements were resolved by consulting a third reviewer. Studies of low quality based on the JBI assessment of several criteria were included in the results, but with descriptions of their weaknesses. The studies were assessed based on their design, implementation, and analytical features rather than on a specific numerical score, following the PRISMA 2020 statement (Page et al., 2021).

### Synthesis Methods

Given the diverse nature of the included studies (in terms of countries of origin, study settings, samples, methods, measures and data analyses, outcomes, and emphases on confounding variables), the results were synthesized into a narrative summary. The findings of the included studies were synthesized and structured based on ownership type, psychosocial work environment variables, and various well-being outcomes. Findings were discussed following the PRISMA guidelines (Page et al., 2021). Given that Muntaner et al. (Muntaner et al., 2004, 2011, 2015; Muntaner, Li, et al., 2006; Muntaner, Van Dussen, et al., 2006) used the same dataset in several studies, their studies were jointly evaluated based on the sample, rather than as different studies. Similarly, the Finnish studies (Heponiemi et al., 2011, 2012a, 2012b) also used the same dataset but with different outcomes for each study.

## Results

The characteristics, demographic groups, and statistical results with outcomes for each study were extracted into two tables, which have been deposited in the Open Science Framework: https://osf.io/wgn3c/?view_only=acef1f491cc1457cad937fd54a5 395aa.

### Study Selection

**Figure 1.**
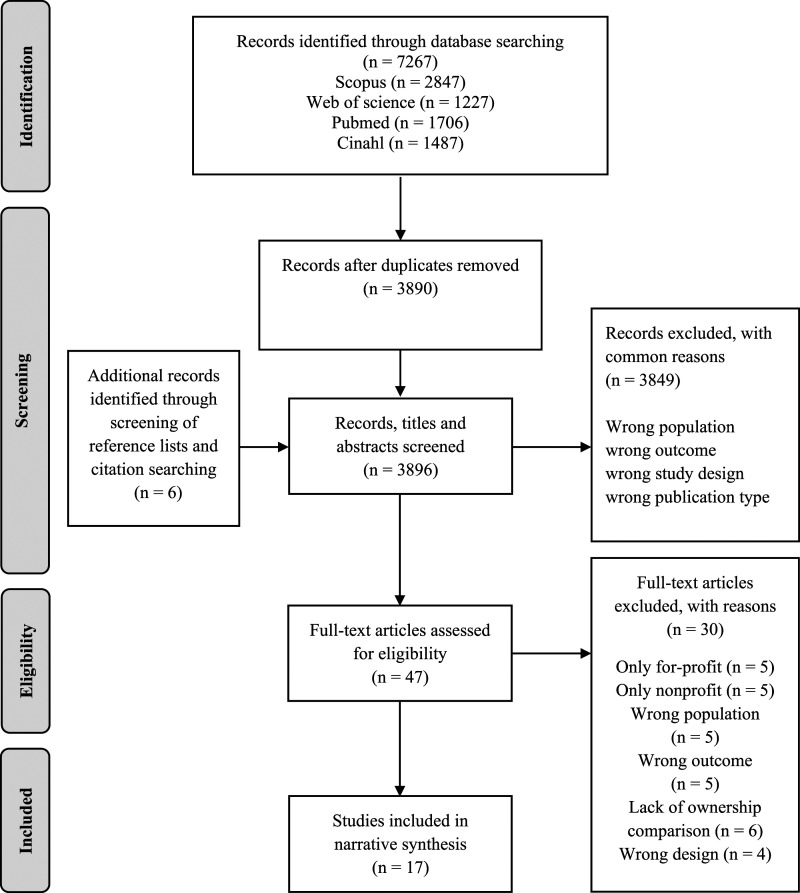
Flow chart of screening and selection process for the included articles.

For the number of studies screened, assessed for eligibility, and included in the review, with reasons for exclusion, see the PRISMA flow diagram (Moher et al., 2009) in Figure 1. In total, 3890 records were identified after the search. After screening, 47 articles were assessed for eligibility, 16 of which were included in the synthesis. Six additional records were included after examining the reference lists of the articles assessed for eligibility and one of these was included, leading to a total of 17 included articles.

### Study Characteristics

The studies were published between 2004 and 2017 and included 12,843 participants. Six studies were from Europe: Switzerland (Graf et al., 2016), Sweden (Höckertin, 2008), Germany (Wendsche et al., 2016), and Finland (Heponiemi et al., 2011, 2012a, 2012b). Nine were from the United States (Choi et al., 2012; Decker et al., 2009; Filipova, 2011; Hamann & Foster, 2014; Muntaner et al., 2004, 2011, 2015; Muntaner, Li, et al., 2006; Muntaner, Van Dussen, et al., 2006). The remaining two articles were from Israel (Iecovich & Avivi, 2017) and Taiwan (Chen et al., 2015). One study included longitudinal data (Muntaner, Li, et al., 2006), while the rest were cross-sectional. Eight of the articles used ownership type as an independent variable, while the others measured it as a confounding variable or covariate in the analysis. The number of participants ranged from 152 to 5323. The studies included nurses with different qualifications, that is, registered nurses (RNs), licensed practical nurses (LPNs), and certified nursing assistants (CNAs).

In total, six psychosocial work environment variables and ten well-being variables were identified; the most common outcome was job satisfaction. However, most articles used different scales for similar outcomes.

The following studies were considered for inclusion but were excluded for the following specific reasons: (Ren, 2013)—the main variable, value congruence, was not considered to represent work well-being or part of the psychosocial work environment; (Islam et al., 2017)—most of the sample comprised managers who could not be distinguished from the other staff; (Noelker et al., 2009)—compared nursing homes with home care and ownership status separately, without any interaction effects reported; and (Gaudenz et al., 2019)—three ownership types were grouped in an adjusted turnover intention model, without data to investigate the possible effect of ownership.

### Risk of Bias

The JBI cross-sectional appraisal checklist was used for all studies (Moola et al., 2020). The checklist contained eight questions evaluating overall article quality. Three additional questions from the JBI checklist for cohort studies that evaluated the longitudinal reliability of the study were considered for the study with longitudinal data (Muntaner, Li, et al., 2006). The risk of bias analysis is presented in Table 2. Common issues identified were the lack of detailed setting descriptions and clear inclusion criteria. Furthermore, some studies used self-constructed measurement variables for the exposure and/or outcome. These measures were not always in English, rendering them difficult to assess. Some studies did not analyze the effect of ownership when considering confounding variables. The specific risk of bias was considered and noted in the synthesis of results.

**Table 2.** Risk of bias assessment using the Joanna Briggs Institute Appraisal Tools for the included studies (*n* = 17).

Main Author and year of Publication	Clear Inclusion Criteria	Detailed Setting Description	Valid/Reliable Exposure	Objective Standard Measurement Criteria	Confounding Factor Identified	Strategies for Confounding Factors	Valid/Reliable Outcome	Appropriate Statistical Analysis
Chen et al., 2015	Yes	Yes	Unclear	*n*/a	Yes	Yes	Unclear	Yes
Choi et al., 2012	No	Yes	Yes	*n*/a	Yes	Yes	Yes	Yes
Decker et al., 2009	Yes	Yes	Yes	*n*/a	Yes	Yes	Unclear	Yes
Filipova, 2011	Yes	No	Yes	*n*/a	Yes	Yes	Yes	Yes
Graf et al., 2016	Yes	Yes	Yes	*n*/a	Yes	Yes	Yes	Unclear
Hamann & Foster, 2014	No	No	Yes	*n*/a	Yes	Yes	Yes	Yes
Heponiemi et al., 2011	Yes	Unclear	Yes	*n*/a	Yes	Yes	Yes	Yes
Heponiemi et al., 2012a	Yes	Unclear	Yes	*n*/a	Yes	Yes	Yes	Yes
Heponiemi et al., 2012b	Yes	Unclear	Yes	*n*/a	Yes	Yes	Unclear	Yes
Höckertin, 2008	No	Unclear	Yes	*n*/a	No	No	Yes	Yes
Iecovich & Avivi, 2017	Yes	No	Yes	*n*/a	Yes	No	Yes	Yes
Muntaner et al., 2004	No	No	Yes	*n*/a	Yes	Yes	Yes	Yes
Muntaner et al., 2011	No	No	Yes	*n*/a	Yes	Yes	No	Unclear
Muntaner et al., 2015	No	No	Yes	*n*/a	Yes	Yes	Yes	Yes
Muntaner, Li, 2006	Yes	Yes	No	*n*/a	Yes	Yes	Unclear	Unclear
Muntaner, Van Dussen, 2006	Yes	Yes	Yes	*n*/a	Yes	Yes	Yes	Unclear
Wendsche et al., 2016	No	Yes	Yes	*n*/a	Yes	Yes	Yes	Yes

*Note.* n/a = not applicable. The assessment of objective standard measurement criteria is to determine if patients were included based on a specified diagnosis or definition, which was deemed not applicable considering the aim of the review. Unclear was selected as an assessment if, for example, the study used newly self-constructed measurement variables, included the study setting but not for ownership information, or if the statistical analysis lacked some information to fully understand the authors’ interpretations.

### Synthesis of Results

**Figure 2.**
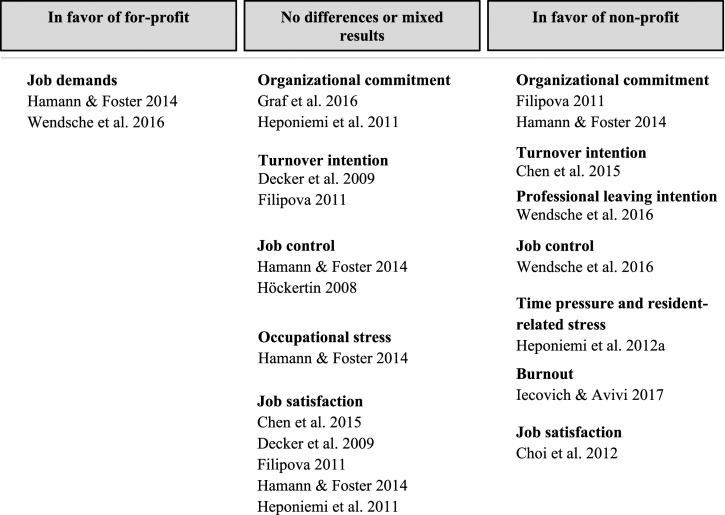
A simplified summary of findings of the main outcomes (at least measured in two study samples) between profit orientation in nursing homes.

The results were structured based on the outcomes of the included studies concerning the psychosocial work environment and well-being of direct-care staff. There were a few country-specific variations. Staff mix and country were noted for each study. A simplified summary of the findings is presented in Figure 2.

#### Psychosocial Work Environment Variables and Profit Status

The following outcomes were identified from the included studies as psychosocial work environment variables: job demands (Hamann & Foster, 2014; Wendsche et al., 2016), job control (Hamann & Foster, 2014; Höckertin, 2008; Wendsche et al., 2016), social support (Hamann & Foster, 2014), job involvement (Heponiemi et al., 2011), team climate with sub-dimensions, role ambiguity and role conflict (Heponiemi et al., 2012b), and job participation (i.e., influence on workplace decisions) (Höckertin, 2008).

Hamann and Foster (2014) investigated different U.S. ownership types concerning job demands, job control, and social support. The study recognized workload as equivalent to job demands. Ownership type had no impact on job control or social support. However, ownership was significant for job demands, and CNAs and RNs working in the public/non-profit sector experienced higher work demands than for-profit staff (non-significant for LPNs). Similarly, Wendsche et al. (2016) found that German RNs reported higher job demands in public/non-profit care than in for-profit care and in nursing homes than in home care; for-profit RNs reported less job control than their non-profit counterparts. Höckertin, 2008 investigated job control and job participation in Swedish nursing homes and obtained inconsistent results, with no significant difference between public and private staff, but that non-profit (i.e., cooperative) nursing home staff perceived higher job control and job participation than did either public or private staff. Notably, that study had an unclear description of the study setting, with unspecified staff mix, and confounders were not considered.

Studies in Finland by Heponiemi et al. (2011, 2012b) included LPNs and RNs, but differences in the results depending on staff mix were not specified. They found higher degrees of role ambiguity among for-profit staff than their non-profit counterparts. There were no significant differences in terms of role conflict and profit orientation. There were no differences between ownership types and job involvement (Heponiemi et al., 2011). Heponiemi et al. (2012b) also examined associations between ownership type and perceived team climate for nurses in Finnish sheltered houses and nursing homes. Although sheltered homes are not synonymous with nursing homes, the studies were included after email contact with the authors, who explained that staff in Finnish sheltered homes work inside the facilities providing 24-hour assistance. Team climate measured items concerning participative safety, support for staff member innovation, vision (i.e., consideration of staff ideas), and task orientation. The dimensions of team climate were higher in non-profit sheltered homes than in for-profit or public sheltered homes.

#### Well-Being: Organizational Commitment and Profit Status

Two European and two U.S. studies investigated forms of organizational commitment. No clear differences in organizational commitment were found in the European studies, while both U.S. studies indicated higher commitment in non-profit than for-profit nursing homes. Graf et al. (2016) found that RNs, LPNs, CNAs, and nurse aides in Swiss public, full private ownership, and private subsidized nursing homes differed in affective organizational commitment; staff in publicly owned nursing homes had a slightly lower mean value. Higher education levels were associated with higher commitment. However, the result of organizational commitment had a minimal effect size. Heponiemi et al. (2011) found that organizational commitment was lower in for-profit sheltered homes than non-profit nursing homes in Finland when perceived organizational justice was low, but that there was no difference when justice levels were perceived as good.

Filipova (2011) found that U.S. RNs and LPNs’ organizational commitment was stronger in non-profit and government-controlled nursing homes than in for-profit skilled nursing facilities. Hamann and Foster (2014) found that U.S. LPNs and CNAs in public nursing homes reported higher organizational commitment than their for-profit counterparts while controlling for workload (non-significant for RNs).

#### Well-Being: Job Satisfaction and Profit Status

There was no conclusive evidence that staff under either ownership type had better self-rated job satisfaction. One study found that job satisfaction was lower for U.S. RNs, LPNs, and CNAs in for-profit nursing homes (Choi et al., 2012), another found no difference in overall job satisfaction depending on profit orientation for RNs, LPNs, and CNAs (Hamann & Foster, 2014), and the other four had conflicting results.

Filipova (2011) found that job satisfaction was slightly higher for RNs and LPNs in for-profit than non-profit nursing homes. There were no differences between government-controlled and for-profit homes. Decker et al. (2009) measured for-profit ownership as a covariate of intrinsic job satisfaction and overall job satisfaction for U.S. CNAs. For-profit was associated with less self-rated intrinsic job satisfaction than non-profit ownership, but there were no differences in overall job satisfaction. One risk of bias concern was the use of a self-constructed single-item measure of job satisfaction. One Taiwanese study by Chen et al. (2015) compared extrinsic/intrinsic job satisfaction among RNs and LPNs in private for-profit and private non-profit nursing homes. There were no differences in either dimension. Heponiemi et al. (2011) found that Finnish nurses’ job satisfaction was lowest among those who worked in for-profit homes and had low levels of perceived organizational justice; there were no differences when justice levels were high.

#### Well-Being: Depression, General Health, and Profit Status

Muntaner et al. (Muntaner et al., 2004, 2011, 2015; Muntaner, Li, et al., 2006; Muntaner, Van Dussen, et al., 2006) investigated depressive disorder and symptoms, and general health among 868 CNAs. Data were collected from 1999 to 2002 for five publications. Two of three studies found associations between profit status and self-reported depressive disorder and symptoms (Muntaner et al., 2004, 2015), with for-profit ownership being associated with a higher risk of depressive disorder and symptoms. Muntaner, Li, et al. (2006) found that when controlling for the Gini index and proportion of African Americans living in the county, type of ownership was no longer significant for depressive disorder. One notable risk of bias was the mix of longitudinal and cross-sectional data without providing sufficient information concerning the setting and data usage in the analyses. Another study (*n* = 395) found no difference between profit orientations for depressive disorder or symptoms (Muntaner, Van Dussen, et al., 2006). Muntaner et al. (2011) investigated overall staff health using a single-item question. The result indicated that for-profit nursing home staff were more likely to report poor health; however, the study lacked information concerning the health measure and study setting.

#### Well-Being: Intention To Leave the Unit and Profession, and Profit Status

Four studies investigated relationships between intention to leave either the unit or the profession and profit status. Most of these studies found higher intentions to leave among for-profit than non-profit nursing home staff. Wendsche et al. (2016) found that German RNs in for-profit nursing homes had higher intentions to leave their profession than their counterparts in public/non-profit nursing homes. Chen et al. (2015) investigated RN and LPN turnover intention in Taiwan, where they divided staff into three groups with low, medium, and high turnover intentions. For-profit ownership was a significant factor when comparing the high- and low-turnover-intention groups. However, the sample size was small (*n* = 186), and only 28% of the included staff worked in non-profit nursing homes. Filipova (2011) obtained inconsistent results: U.S. LPNs in government facilities had lower intentions to leave than those in for-profit facilities, but there were no differences between non-profit and for-profit LPNs. Decker et al. (2009) found no significant results between nursing home ownership type and intention to leave the unit for U.S. CNAs. However, there was a risk of bias due to the use of a self-constructed single-item scale, which the authors did not specify or justify.

#### Well-Being: Burnout, Occupational Stress, and Profit Status

The studies were diverse in their measurement of stress and burnout syndromes and had some methodological issues. For example, Iecovich and Avivi (2017) investigated ageism and burnout in long-term care in Israel. LPNs and RNs working in a for-profit facility reported greater overall work burnout than did the non-profit staff. For-profit status had the highest impact of all the included variables in explaining variance in burnout. Hamann and Foster (2014) found no significant difference in occupational stress among RNs, LPNs, and CNAs working in public/non-profit versus for-profit nursing homes. The authors claimed that occupational stress was higher in the public/non-profit sector than in the for-profit sector; however, they used a *p*-value of .10. Heponiemi et al. (2012b) explored relationships between profit status and work-related stress in the form of time pressure and resident-related stress. Staff working in for-profit sheltered homes were more sensitive to time pressure and resident-related stress than staff in non-profit homes (education levels unspecified).

#### Well-Being: Other Relevant Outcomes

Heponiemi et al. (2012a) also investigated associations between ownership type and job insecurity or worries about job stability among RNs, LPNs, and care staff without specialized education. Their results indicated that job insecurity and worry about job stability were highest in non-profit sheltered homes. Lower education levels were associated with higher levels of worry about job stability, but strong leadership and fair management mitigated both the insecurity and worries of non-profit sheltered home staff.

## Discussion

This systematic review examined the psychosocial work environment and well-being of direct-care staff under different nursing home ownership types. In terms of psychosocial work environment, job demands, and job control were the only identified factors investigated in more than one study regarding their relationship with nursing home profit status. The most common well-being variables identified were job satisfaction, organizational commitment, and turnover intention. Most of the outcomes slightly favored non-profit over for-profit settings, for example, regarding commitment, stress, and leaving intentions. Job stability, team climate, depressive disorder indicators, and role ambiguity favored non-profit nursing homes; however, these outcomes were only evaluated in one study or sample each, limiting the possibility to generalize. Job demands and job control were higher in non-profit nursing homes; and results for job satisfaction were mixed.

The results regarding profit incentives were similar to those of previous systematic reviews concerning quality (Comondore et al., 2009; Herrera et al., 2014; Xu et al., 2013), staffing levels, and job benefits (Bos et al., 2017), and working conditions (Vrangbæk et al., 2015), that is, slightly in favor of non-profit nursing homes. Bos et al. (2017) argued that work well-being was better in non-profit nursing homes, but their results were limited to the United States and primarily emphasized staffing levels and resident well-being. Similarly, a rapid review found a relationship between for-profit incentives and increased COVID-19 cases and the number of deaths among nursing home residents (Kruse et al., 2021).

Our results concerning job satisfaction were also in line with a recent systematic review, in which, ownership type was not clearly related to RNs’ and LPNs’ job satisfaction in nursing homes (Aloisio et al., 2021). One study in our review by Iecovich and Avivi (2017) showed that for-profit settings were associated with and predictive of overall burnout. Although their sample size was small (*n* = 154), this finding is notable, considering that burnout syndrome is a frequent problem facing nursing home care aides (Cooper et al., 2016) and nursing home staff in general (White et al., 2019, 2020). Since for-profit incentives have been linked to reduced staffing levels and other deficiencies (Harrington et al., 2012; Meagher et al., 2013), it may also be linked to factors that increase burnout risk among nursing home staff. Although job demands were higher in non-profit nursing homes (Hamann & Foster, 2014; Wendsche et al., 2016), job control was also higher (Wendsche et al., 2016), which could indicate that high demands were not necessarily problematic given decent job control, as hypothesized in, for example, the job demands-resources theory (Bakker & Demerouti, 2017).

The synthesized results may also be considered aside from the ownership question: high job demands (Hamann & Foster, 2014; Wendsche et al., 2016), high turnover intentions (Chen et al., 2015; Wendsche et al., 2016), and staff displaying depression and burnout syndromes (Iecovich & Avivi, 2017; Muntaner et al., 2004, 2015)—to name but a few problems—all indicated problems facing direct-care staff in nursing homes in general. Poor work environment and poor work well-being have furthermore been linked to the quality of care (Engström et al., 2021; Hsu et al., 2016; Lundgren et al., 2020; White et al., 2019). These findings, together with the global shortage of healthcare workers projected for 2030 (Liu et al., 2017) and an aging global population (OECD, 2020), are troubling for nursing homes, staff, and residents alike.

Although inconsistent, our findings together with those of previous reviews and studies concerning for-profit incentives in nursing homes should raise concerns among policymakers. Country-based differences should be considered, as it is unlikely that one solution will work everywhere. The Nordic countries have somewhat limited the growth of for-profit ownership, while in North America, for example, most nursing homes are already for-profit owned, a situation unlikely to change soon (Harrington et al., 2017; Meagher et al., 2013). One suggestion in previous reviews was that stakeholders must implement more clearly defined precautionary routines and regulations that address for-profit incentives and risk-related outcomes such as decreased staffing levels or job resources (Comondore et al., 2009; Herrera et al., 2014; Ronald et al., 2016). As discussed by Banerjee et al. (2021), enforcing stricter staffing-level regulations to combat incentives to cut the workforce could be one option. Kruse et al. (2021) discussed different approaches: disincentivizing for-profit ownership, favoring non-profit ownership, and demanding higher minimum staffing standards. Another suggestion has been to demand transparency through reporting on financial and quality outcomes and on how resources are utilized, with the possible addition of regulated cost controls (Harrington et al., 2017; Ronald et al., 2016). For example, the Netherlands screens private providers and restricts profit distribution, and includes strict conditions in procurement documents that all municipalities must follow when subcontracting nursing homes (European Commission; Directorate-General for Employment; Social Affairs and Inclusion, 2021). Although nursing home ownership structures differ internationally, better-regulated staffing levels and higher transparency in how for-profit nursing homes utilize their resources are two actions that may be considered no matter the country. This is also related to the longstanding workforce crisis in long-term care with difficulties in filling vacant jobs, retaining labor, and retaining qualified staff with staff shortages as a result (European Commission; Directorate-General for Employment; Social Affairs and Inclusion, 2021; Scales, 2020, 2021). Improving the work environment may enhance job attractiveness and help nursing homes retain and hire staff to help deal with these challenges. Policymakers must consequently consider the work environment of the direct-care staff when establishing quality of care policies for older adults, given that this is in the interest of both staff and residents.

### Limitations

To our knowledge, this systematic review was the first to compile and synthesize international results concerning the relationship between nursing home ownership status and the psychosocial work environment and well-being of direct-care staff. However, this meant that the evidence was difficult to generalize due to differences between countries, staff mix, and study settings. The study divided ownership according to profit incentives, somewhat oversimplifying the situation. Differences between private providers concerning, for example, chain affiliation, corporate-specific strategies, and size of facilities also matter in addition to ownership (Harrington et al., 2017; Hsu et al., 2016). Another limitation was the language filter. The review used a thorough methodology through the PRISMA guidelines (Moher et al., 2009; Page et al., 2021) and the JBI quality appraisal tools (Briggs, 2014). Furthermore, all search strategies were developed in collaboration with an academic information specialist. Additionally, a wide search strategy was utilized, allowing variables that might otherwise have been missed to be included. However, the certainty of the evidence is limited due to the vast differences in outcome variables, the inconsistent or conflicting results for some outcomes, and the fact that most of the studies only included cross-sectional data. The number of longitudinal studies in the area is likely limited, since searching multiple databases identified only one such study. Furthermore, to minimize errors, the review process involved two different reviewers for screening, full-text reading, and bias risk assessment. Additionally, some authors of the included studies were contacted to enhance the reliability of the results.

## Conclusions

This systematic review contributed to building the knowledge of nursing home profit orientation in general, while also compiling results concerning the psychosocial work environment and well-being of direct-care staff under different nursing home ownership types. Although the results were inconsistent, the overall results combined with those of previous research indicated that for-profit ownership of nursing homes might have adverse consequences for both staff and residents. This review has further identified a potential research gap in terms of longitudinal studies of the relationship between for-profit ownership status and the psychosocial work environment in nursing homes, with confounders considered. Overall, the results accentuated the role that profit incentives may have on the psychosocial work environment and well-being of nursing home staff.

## Supplemental Material

Supplemental Material - Psychosocial Work Environment and Well-Being of Direct-Care Staff Under Different Nursing Home Ownership Types: A Systematic Review Click here for additional data file. Supplemental Material for Psychosocial Work Environment and Well-Being of Direct-Care Staff Under Different Nursing Home Ownership Types: A Systematic Review by Tomas Lindmark, Maria Engström, and Sven Trygged in Journal of Applied Gerontology
